# The effect of Crocin on sperm parameters disorders induced by peripheral administration of nitrosamines in male rats

**DOI:** 10.25122/jml-2019-0144

**Published:** 2022-03

**Authors:** Mohammad Reza Salahshoor, Paria Azari, Shiva Roshankhah, Abdolhamid Zokaei, Azadeh Foroughinia

**Affiliations:** 1. Department of Anatomical Sciences, Medical School, Kermanshah University of Medical Sciences, Kermanshah, Iran; 2. Cardiovascular Research Center, Kermanshah University of Medical Science, Kermanshah, Iran

**Keywords:** Nitrosamine, reproductive parameters, Crocin, DNA – Deoxyribonucleic Acid, FBS – Fetal bovine serum, WHO – World Health Organization, H&E – Hematoxylin and eosin dry, ROS – Reactive oxygen species, cAMP – Cyclic AMP, adenosine 3',5'-cyclic monophosphate, TAC – Total antioxidant capacity

## Abstract

Nitrosamines are carcinogenic agents which can unfavorably affect some male reproductive parameters. Humans are exposed to nitrosamines through various routes, the most important of which is the diet. Crocin is a carotenoid and is accountable for the red color of saffron. Crocin has numerous pharmacological actions, such as antioxidant roles and radical scavenging. The aim of this study was to evaluate the effects of Crocin against Nitrosamine – induced damage to the reproductive parameter of male rats. In this experimental study, 48 male rats were randomly assigned to 8 groups: control normal and Nitrosamine control groups (40 mg/kg); Crocin (12.5, 25, and 50 mg/kg) and Nitrosamine + Crocin (12.5, 25, and 50 mg/kg). Treatments were administered intraperitoneally and gavaged daily for 28 days. The sperm parameters, total antioxidant capacity, testosterone level, and seminiferous tube diameter were assessed. Nitrosamine significantly decreased sperm parameters (p<0.001). The Crocin and Crocin + Nitrosamine treatments at complete doses significantly improved all parameters (p<0.001). Crocin compensated for the toxic effect of Nitrosamine on reproductive parameters.

## INTRODUCTION

Sterility in men affects a large part of the population [1]. Nitrosamines result from the reaction between nitrite, free amino acids, and amines [2]. Humans are exposed to nitrosamines through various routes, especially food (including meat). During this process, nitrite reacts with secondary amines in meat and produces nitrosamines [3]. Nitrate received through food or water can be made through the internal synthesis of nitrate, the introduction of nitrate from blood to saliva, the change of nitrate through bacteria in the saliva, and the modification of nitrite in the blood [4]. Human contact with nitrosamine compounds is associated with the risk of esophageal, gastric, and bladder cancer [5]. Nitrosamines are widely found in nourishments and natural harvests, reproduction materials, chemicals, cigarettes, solvents, medicines, leather and plastics products, and cosmetics [6].

Furthermore, nitrosamines can induce DNA damage by forming highly reactive diazonium ions. Nitrosamines automatically apply their destructive effects by forming diazonium and oxonium ions and alpha-hydroxylation metabolites by activating the cytochrome P-450 enzyme [7]. In addition, nitrosamines cause oxidative stress and create free radicals in the body, resulting in cellular damage [8]. Saffron is a member of the Crocus sativus L. family with various therapeutic effects due to the available biochemicals [9], which has been used to treat infertility [10]. Crocin has numerous pharmacological actions such as antioxidants, anticancer, radical scavenging, and genoprotective effects [11]. This experimental investigation analyzed the effect of Crocin on changes in reproductive parameters after induction of injury following administration of Nitrosamine in male rats.

## MATERIAL AND METHODS

### Laboratory rats

48 rats (Wistar race, male, 220-250 gr) were obtained from the Pasteur Institute. All standard conditions necessary for animal growth were provided, including physiological photocycle and temperature [11].

### Animal groups

The rats were grouped (n=8) as 1^st^ normal control group (positive control) which received normal saline by gavage and injection, 2^nd^ control group of Nitrosamine (negative control) which received 40 mg/kg single intraperitoneal dose Nitrosamine (6); in the 3^rd^–5^th^ Crocin groups each animal orally received 12.5, 25, and 50 mg/kg of Crocin for 28 days. 6^th^–8^th^ Nitrosamine + Crocin groups received a single dose of 40 mg/kg Nitrosamine followed by 12.5, 25, and 50 mg/kg Crocin orally for 28 days [9].

### Tissue sample collection

Animals were anesthetized using ether following treatment completion. After thoracotomy, 5cc of blood sample was aspirated from the left ventricle of the heart and transferred into anticoagulant test tubes. Centrifuging was conducted (3000 rpm, 15 min), and the blood serum was isolated for total antioxidant capacity, nitric oxide, and testosterone levels measurements. Then, the epididymis tail was dissected in DMEMF12/FBS5% culture medium, and the testes were fixed in a 10% formalin for histopathological assessments [12].

### Sperm collection

Both cauda epididymidis of a single animal were cut in a warmed petri dish with condition medium (10 ml Hank's balanced salt solution) at 37°C. After 15 min, the suspension was slightly shaken to normalize cell dispersion [12].

### Progressive motility

Sperm motility was grouped according to the guidelines approved by the World Health Organization (WHO): a) quick progressive motility in a direct line, b) slow progressive motility in direct or indirect line, c) no progressive motility, and d) no motility [1]. Class a (quick progressive motility) was examined in this study. Sperms suspended in culture medium were aspirated (50 μl) and examined microscopically.

### Survival rate

To assess the survival rate of sperms, the eosin was used to stain dead sperms, distinguishing them from the living cells. 20 μl of the sample was mixed with 20 μl eosin. 2–5 min later, 10 μl of the mixture was located on a neobar slide and examined microscopically (40×). 100 sperm cells were calculated from each random sample from the 10 fields of imagining, and the percentage of live sperm cells was documented [9].

### Normal morphology of sperms

All sperm smears collected from the right cauda epididymis were examined morphologically. For this purpose, the eosin/nigrosin was used to stain the sperms and assess the normal spermatozoa morphology using a light microscope (400× magnification) [12].

### Calculation of sperm count

Sperm solution (15 μL) was transferred to a hemocytometer located in a Petri dish. This solution was stabled for 10 min for high-quality cell counting. The counting procedure was applied microscopically per 250 small squares of hemocytometer (40x). Finally, the count of sperm was calculated per mm^3^ (sperm count×dilution/number counted in mm^2^ the depth of the chamber) [13].

### The germinal layer of seminiferous tubules

To assess the histopathological changes of seminiferous tubules, the fixed testes samples were treated on tissue processing, including dehydration, clearing, and embedding. Serial sections (5 μ) were stained using H&E (30 sections from each block). At least 10 tubules from each segment were assessed in each testis. The height and the diameter of germinal layers were measured using a Motic camera and software (Moticam 2000; Spain) [13].

### TAC measurement

An associated biochemical kit (Cat No: TAC-96A, ZellBioGmbH-Germany) was purchased. The reaction of this kit is based on the oxidation colorimetric resuscitation. This kit contained 1 reagent ready to use, buffer X 200, dye powder, reaction suspension solution, standard, and a microplate of 96 wells. The sensitivity of the kit was equal to 0.2 mm, and final absorbance was read at 540 nm [6].

### Testosterone

Previous serum samples (stored at -196°C) were used for serum testosterone level assessment using ELISA (Abcam 108666, USA) technique [1].

### Statistical analysis

One-way analysis of variance (one-way ANOVA) was used for statistical analysis, and Tukey post hoc test was used to determine the difference between the groups. SPSS 16 was used for data analysis, and the results were expressed as mean±standard error, and p<0.05 was considered significant.

## RESULTS

### Sperm motility and viability

Both progressive motility and viability of sperms were significantly reduced following Nitrosamine administration compared to the normal control group (p<0.001). Furthermore, these indices were significantly accelerated in all treated Crocin and Nitrosamine + Crocin groups compared to the Nitrosamine control group (p<0.001) (Table 1).

**Table 1.** Different sperm parameters between treatment groups in Balb/c mice.

Groups Parameters	Normal control	Nitrosamine control	C 12.5mg/kg	C 25mg/kg	C 50mg/kg	C/N 12.5mg/kg	C/N 25mg/kg	C/N 50mg/kg
**Normal morphology (%)**	81.81±3.6	50.45±4.7 *	82.98±5.6 †	83.1±4.1 †	81.32±36 †	61.71±4.3 ^#^	67.03±2.4 ^#^	70.93±5.1 ^#^
**Count (106)**	3.78±0.07	0.81±0.06 *	3.55±0.03 †	3.05±0.16 †	2.9±0.09 †	1.56±0.07 ^#^	1.9±0.03 ^#^	1.99±0.06 ^#^
**Motility (%)**	72.83±6.1	25±2.1 *	70.83±0.9 †	69.83±4.4 †	71.83±3.5 †	52.5±2.3 ^#^	56.33±4.7 ^#^	61.3±5.1 ^#^
**Viability (%)**	81.91±6.1	44.36±3.4 *	82.84±2.3 †	83.49±4.1 †	81.11±5.8 †	66.96±4.8 ^#^	66.36±3.3 ^#^	71.05±5.1 ^#^

One-way ANOVA. Data are presented as mean±SEM. * – P<0.05 compared to the normal control group. † – P<0.05 compared to the Nitrosamine control group. ^#^ – P<0.05 compared to the Nitrosamine control group. C – Crocin; C/N – Crocin + Nitrosamine.

### Count and normal morphology of sperms

These features were significantly reduced in the Nitrosamine control group compared to the normal control group (p<0.001) and enhanced in whole treated Crocin and Nitrosamine + Crocin groups compared to the Nitrosamine control group (p<0.001) (Table 1).

### The germinal layer of seminiferous tubules

**Figure 1.**
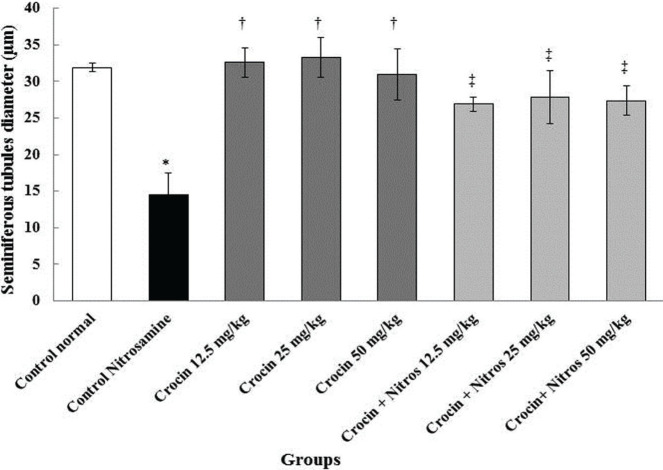
Comparison of diameter of seminiferous tubule in treatment groups. * – Significant decrease compared to normal control group (p<0.001). † – Significant compared to the Nitrosamine control group (p<0.001). ‡ – Significant compared to the Nitrosamine control group (p<0.001).

**Figure 2.**
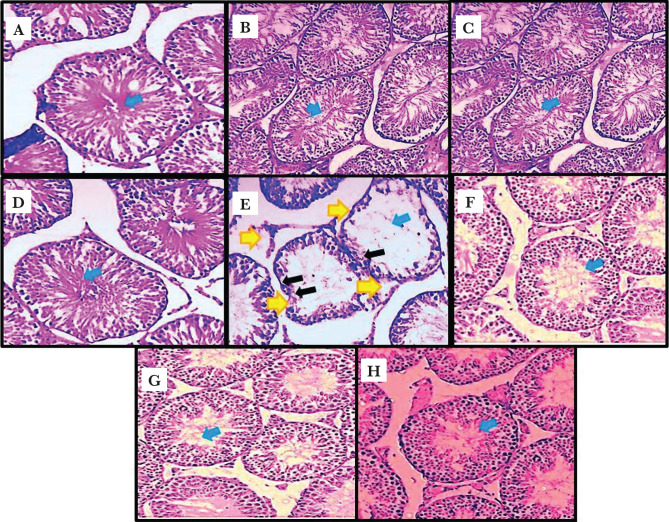
The effect of Nitrosamine, Crocin and Crocin + Nitrosamine on seminiferous tubules (magnification 40×). Normal seminiferous tubule structure was observed in normal control group (A); Crocin groups (12.5 mg/kg) (B); (25 mg/kg) (C); (50 mg/kg) (D) and Nitrosamine + Crocin groups (12.5 mg/kg) (E) – the black arrow identifies the germinal layer (reduction in epithelial height and irregularities in the structure of the margin of tubules), the yellow arrow identifies the destruction of membrane seminiferous tubules structure and the blue arrows identify sperm cells; (25 mg/kg) (F); (50 mg/kg) (G); a decrease in the germinal layer of seminiferous tubules, germinal layer and sperm cells was observed in the Nitrosamine control group (H).

The diameter and height of the germinal layer were significantly reduced (p<0.001) in the Nitrosamine administration groups compared to the normal control group (p<0.001). These characteristics were also significantly increased (p<0.001) in animal groups treated with Crocin and Nitrosamine + Crocin than the Nitrosamine control group (p<0.001) (Figures 1 and 2 A–H).

### Total antioxidant capacity

**Figure 3.**
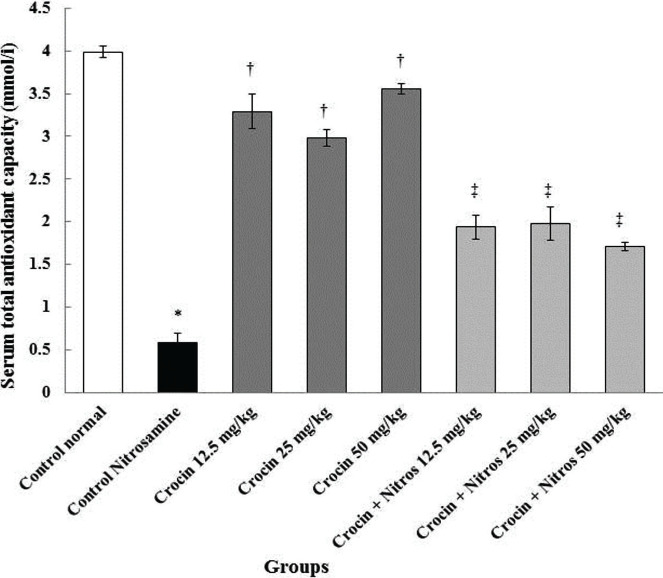
Comparison of total antioxidant capacity in Nitrosamine and normal control and Crocin and Crocin + Nitrosamine groups. * – Significant increase compared to normal control group (p<0.001). † – Significant increase compared to the Nitrosamine control group (p<0.001). ‡ – Significant increase compared to Nitrosamine control group (p<0.001).

TAC level was significantly decreased (p<0.001) in the Nitrosamine control group compared to the normal control group (p<0.001). Also, Crocin increased TAC value in Crocin doses compared to Nitrosamine control groups (p<0.001). This value was also significantly improved in all Nitrosamine + Crocin groups than the Nitrosamine control group (p<0.001) (Figure 3).

### Testosterone

**Figure 4.**
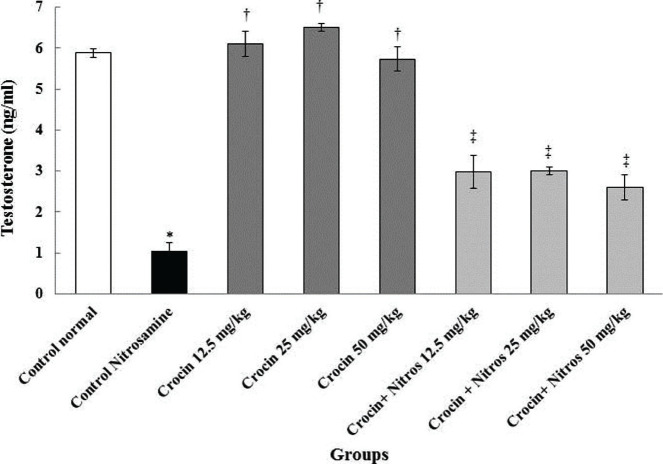
Comparison of testosterone hormone level in treatment groups. * – Significant increase compared to normal control group (p<0.001). † – Significant increase compared to the Nitrosamine control group (p<0.001). Significant increase compared to Nitrosamine control group (p<0.001).

In this study, Nitrosamine control group was compared with normal control group and other groups. Nitrosamine significantly reduced testosterone levels (p<0.001) compared to the normal control group. Also, testosterone levels were significantly increased (p<0.001) in whole Crocin and Nitrosamine + Crocin groups than Nitrosamine control (Figure 4).

## DISCUSSION

The antioxidant system protects the human body from free radicals. Oxidative stress is caused by a mismatch between the amount of free radicals produced and the antioxidant capacity of the body [14]. The current study found that nitrosamine treatment lowered the survival rate, quantity, and motility of sperm cells, but crocin administration prevented these variables from dropping. ROS interferes with sperm DNA and RNA production and mitochondrial activity [9].

It is also probable that oxidative stress affects sexual germ cells, in the same way, disrupting their division and differentiation affecting many sperm cells on the base membrane [12]. Khan *et al.*'s findings were consistent with the findings of the current study, which showed that arsenic-induced oxidative stress in male rats resulted in ROS production, sperm deformity, significant reductions in motility and number of sperm, and testosterone levels when compared to the control group [15]. The emergence of cell peroxidation within the cell membrane and organelles is the first result of ROS assault on membrane components. Because sperm lost a considerable portion of their cytoplasm during spermatogenesis (due to a lack of antioxidant mechanisms), they appear to be more vulnerable to increased ROS levels in the environment than somatic cells [1]. Compared to the control group, arsenic-induced oxidative stress in male rats caused ROS, sperm deformities, DNA degradation, lower fertility index, motility and quantity of sperm, and testosterone levels [15]. Adeleke *et al.* found that giving N-Nitrosodimethylamine to male rats resulted in a substantial reduction in sperm motility and survival compared to the control group, which supports the findings of this study [16]. Crocin can improve sperm motility by raising intracellular calcium concentrations and cAMP analogs that inhibit phosphodiesterase penetrating the membrane [17]. There is no possibility of oxidative damage regeneration due to the limited number of cytoplasmic enzymes. Antioxidants and antioxidant enzymes are extremely important in the semen fluid to defend against oxidative damage [12]. The results of Griveau *et al.*, which corroborated the findings of this investigation, demonstrated that sperm proximity to Crocin caused non-moving sperm motility [18]. The current study found that Nitrosamine decreased total antioxidant capacity serum levels but that TAC levels improved in the Crocin-treated groups. In this study, the effects of oxidative stress from Nitrosamine on reproductive parameters are demonstrated by a decrease in total antioxidant capacity. In testicular tissue, Nitrosamine causes oxidative stress. The antioxidant volume of testicular tissue can be particularly damaging due to its fast metabolism, large quantities of unsaturated fatty acids in its cell membranes, and cell growth [19].

Crocin's antioxidant and anti-lipid peroxidation activities were highlighted in this study by increased TAC levels in rats treated with the drug. The results of Nermin *et al.* demonstrated that diethylnitrosamine treatment caused hepatocarcinogenesis in male rats, resulting in a substantial reduction in serum total antioxidant capacity, which supports our findings [20]. This study found that nitrosamine administration lowered normal sperm cell morphology, the diameter of seminiferous tubules, and testosterone levels, while Crocin treatment increased normal sperm cell morphology, the diameter of seminiferous tubules, and testosterone levels. By creating reactive oxygen species such as superoxide and hydrogen peroxide, Nitrosamine can cause increased oxidative stress, DNA damage, lipid peroxidation, and the production of new protein compounds [21]. In the nitrosamine control group, it seems that wall cells of the germinal layer of seminiferous tubules rapidly differentiated and released from the tubules' wall, increasing tubule diameter [22]. According to the findings of Mesbahzadeh *et al.*, Crocin treatment dramatically increased the width of the germinal layer of seminiferous tubules and testosterone levels, which is compatible with the findings of this investigation [23]. Crocin's vasodilation and enhanced blood flow, in addition to its antioxidant characteristics, appear to be a factor in enhancing testosterone levels in the current study [9–11].

## CONCLUSION

The findings of this study show that Nitrosamine can negatively affect male reproductive parameters. Moreover, Crocin has an antioxidant and defense impact, increasing the quality of certain spermatozoa and enhancing normal morphology, vitality, motility, and count. Crocin may be used in the treatment of infertile males in order to increase male fertility. Crocin's antioxidant capabilities may be one of the reasons for its favorable reproductive results. Additional research is required to fully understand its complex mechanism of action.

## ACKNOWLEDGMENTS

### Conflict of interest

The authors declare no conflict of interest.

### Ethical approval

This study was approved by the Ethics Committee of Kermanshah University of Medical Sciences (No.IR.KUMS.REC.1396.610).

### Funding

This research was funded by the Research and Technology Deputy of Kermanshah University of Medical Sciences.

### Authorship

AF and MRS contributed to experimental conception and design. PA, SR, AZ, and AF performed the experiments. MRS, AF, and SR analyzed the data. AF, SR, MRS wrote the first draft of the paper. All authors reviewed and approved the final manuscript.
